# The Reform in Spelling

**Published:** 1897-09

**Authors:** 


The Reform in Spelling.—Lanphear, of the *American Journal of Surgery and Gynecology*, is a pioneer in the reform in spelling. He uses as terminal in the participle of many verbs, "t" instead of "ed," and says "trapt," "dresst," "abolisht," "publisht," "overworkt," etc. This is a bold innovation; but one which we hope will become the rule. Next.

